# Potential Involvement of LOX-1 in Functional Consequences of Endothelial Senescence

**DOI:** 10.1371/journal.pone.0020964

**Published:** 2011-06-15

**Authors:** Magomed Khaidakov, Xianwei Wang, Jawahar L. Mehta

**Affiliations:** Department of Medicine, Central Arkansas Veterans Healthcare System and University of Arkansas for Medical Sciences, Little Rock, Arkansas, United States of America; Tor Vergata University of Rome, Italy

## Abstract

Numerous studies have described the process of senescence associated with accumulation of oxidative damage, mutations and decline in proliferative potential. Although the changes observed in senescent cells are likely to result in significant phenotypic alterations, the studies on consequences of endothelial senescence, especially in relation to aging-associated diseases, are scarce. We have analyzed effects of senescence on the functions of endothelial cells relevant to the development of atherosclerosis including angiogenesis, adhesion, apoptosis and inflammation. In the course of progressing through the passages, human umbilical vein endothelial cells (HUVECs) displayed significant increase in size (+36% passage 12 vs. passage 4 , p<0.001) and reduction in both basal and *VEGF*-stimulated tube formation. The analysis of a scavenger receptor *LOX-1*, a key molecule implicated in atherogenesis, revealed a significant decline of its message (mRNA) and protein content in senescent endothelial cells (−33%) and in aortas of 50 wk (vs. 5 wk) old mice (all p<0.01). These effects were accompanied by a marked reduction of the basal expression of *VCAM-1* and *ICAM-1*. Compared to early cultures, late passage HUVECs also exhibited nuclear translocation of *NF-κB* (p65) and reciprocal shifts in *BAX* and *BCL2* protein content resulting in almost 2-fold increase in *BAX/BCL2* ratio and 3-fold increase in apoptotic response to *TNFα* exposure (p<0.04). These changes in senescent endothelial cells are suggestive of aberrant responses to physiological stimuli resulting in a less permissive environment for tissue remodeling and progression of diseases requiring angiogenesis and cell adhesion in elderly, possibly, mediated by *LOX-1*.

## Introduction

Aging is a non-modifiable risk factor for atherosclerosis [1]. However, although the overall consequence of aging is decline in diverse functions resulting in increased susceptibility, the same mechanisms may confer certain advantages by limiting the aggressiveness of disease progression. Although there are difficulties in discerning such phenomena in clinical or epidemiological studies, there is sufficient experimental support for this notion. For example, high cholesterol diet is more atherogenic in the young than in the older mice and rabbits [2], [3].

Endothelial cells play a prominent role in the pathobiology of cardiovascular diseases through their involvement in angiogenesis, cell adhesion/transendothelial migration and inflammatory signaling. Senescence in endothelial cells is characterized by a number of universal features of aging including telomere shortening, accumulation of oxidative damage and deterioration of mitochondrial function [4]–[6]. This process appears to accelerate in regions of the cardiovascular system that are subjected to shear stress and shifts in microenvironment as, for example, within the plaques where oxidized LDL-cholesterol (ox-LDL) selectively accumulates [7]–[9]. Ox-LDL is evolving to be a major determinant of atherosclerotic process. It is taken up by specialized lectin-like scavenger receptor LOX-1. The functional consequences of endothelial senescence, especially in relation to ox-LDL uptake and LOX-1 expression, are not well understood.

The purpose of the present study was to elucidate the effects of senescence on the functions of endothelial cells relevant to atherogenesis.

## Methods

### Reagents and cell lines

Primary human umbilical vein endothelial cells (HUVECs) were purchased from ATCC and cultured in the vascular endothelial growth medium (ATCC) complemented with VEGF endothelial cell growth kit. The cells were cultured for up to 12 passages. For study of senescence, passage 4 (P4), passage 8 (P8) and passage 12 (P12) HUVECs were used.

CJ 57 mice (3 animals per age group) were used for collection of aortas under pentobarbital anesthesia. All animal work was conducted according to relevant local, university, national and international guidelines, and animals were treated humanely (approval by Animal Use Committee of the CAVHS, Little Rock, AR, project number 0018).

### Determination of cell size

Cell size was determined using images taken from passage 4 and passage 12 confluent cultures stained with DAPI. The average cell size was calculated in several images by dividing the area of the field of view on the number of cells (nuclei count).

### Real-Time Quantitative PCR

Quantitative PCR was performed using the Applied Biosystems 7900 real-time PCR system. qPCR specific primers were designed using Probe-Finder (http://www.roche-appliedscience.com) web-based software (Table 1). All qPCR reactions were carried out in a final volume of 15 µl containing 1× of SYBR Green PCR Master Mix (Applied Biosystems, Carlsbad, CA), 300 nM of each gene specific primers, 100 ng cDNA, in sterile deionized water. The standard cycling condition was 50°C for 2 min, 90°C for 10 min, followed by 40 cycles of 95°C for 15 s and 62°C for 1 min. The results were analyzed using SDS 2.3 relative quantification manager software. The comparative threshold cycles values were normalized for GAPDH reference genes. qPCR was performed in triplicate to ensure quantitative accuracy.

**Table 1:** qPCR primers used in this study.

Gene	Accession #	Forward	Reverse
OLR1 (LOX-1)	NM_002543	GCGACTCTAGGGGTCCTTTG	GTGAGTTAGGTTTGCTTGCTCT
VCAM-1	NM_001078	GCTGCTCAGATTGGAGACTCA	CGCTCAGAGGGCTGTCTATC
ICAM-1	NM_000201	TCTGTGTCCCCCTCAAAAGTC	GGGGTCTCTATGCCCAACAA
BAX	NM_138764	GCGTGAAATGGCGTGATCTG	TGAGGCAGGTGAATCGCTTG
Bcl2	NM_000633	GGGGAGGATTGTGGCCTTC	CAGGGCGATGTTGTCCACC
GAPDH	NM_002046	ATGGGGAAGGTGAAGGTCG	GGGGTCATTGATGGCAACAATA

### Western blot

The primary and secondary antibodies were purchased from Santa Cruz Biotechnology (Santa Cruz, CA). Protein was extracted from HUVECs using RIPA buffer (Thermo Fisher Scientific Inc, Rockford, IL). Lysates (∼20 µg of protein) were separated by 10% SDS-PAGE, and transferred to PVDF membrane (Millipore, Bedford, MA). After blocking with PBST containing 5% non-fat milk, membranes were sequentially incubated with primary antibodies (overnight at 4°C), washed with PBST (×3, 10 min each), incubated with HRP conjugated secondary antibodies for 30 min at room temperature, washed, treated with the ECL western blotting substrate (Promega Corporation, Madison, WI) and imaged. The relative expression of proteins was evaluated in relation to β-actin.

### Dil-ox-LDL uptake

For visualization of specific ox-LDL uptake, triplicate cultures of HUVECs in 24-well format were incubated with Dil-ox-LDL 5 µg/ml in presence of 100-fold excess of unlabeled ox-LDL for 2 h at 37°C. After incubation, cells were gently washed with 1× PBS (3 times) and imaged using fluorescent microscopy.

### Immunostaining and fluorescence microscopy

Immunocytochemistry was performed using primary and secondary antibodies (Santa Cruz Biotechnology). Early and late passage HUVECs were grown on 10 mm round cover slips and stained using standard methods. Cells were mounted on microscope slides using Prolong® Gold antifade reagent with DAPI (Invitrogen, Carlsbad, CA) and imaged with LSM510 Zeiss Laser inverted confocal microscope using LSM510 software (Version 4.0 SP1).

### Matrigel tube formation assay

50 µl of matrigel basement membrane matrix (BD Biosciences) was pipetted into each well of a 96-well plate at 4°C and allowed to solidify for 30 min at 37°C. 3×10^4^ HUVECs in 200 µl of complete growth medium were seeded into the wells and incubated overnight. At the end of the experiments, cells were loaded with 10 µM calcein AM (Invitrogen), washed with PBS (×3), and imaged using fluorescence microscopy. The length of tube-like networks was calculated using ImageJ software.

### Detection of apoptosis

Apoptosis was measured using polycaspase assay (ImmunoChemistry Technologies, (Bloomington, MN) according to supplied protocols with minor modifications. Briefly, early and late passage HUVECs were seeded on 10 mm round cover slips, exposed to 1× FLICA reagent and incubated at 37°C, 95% humidity for 60 min. After washing with supplied Wash buffer and fixation, coverslips were mounted on slides using ProLong Gold antifade reagent with DAPI (Invitrogen, Carlsbad, CA) and the percentage of apoptotic cells was quantitated using fluorescent microscopy.

### Statistical analysis

All in vitro experiments have been performed in triplicates. Data were analyzed using Microsoft Excel data analysis package. Comparisons between treated and control groups were made by two-tailed Student's t- test, and a p value<0.05 was considered significant. All results are presented as means ± standard error.

## Results

**Figure 1:**
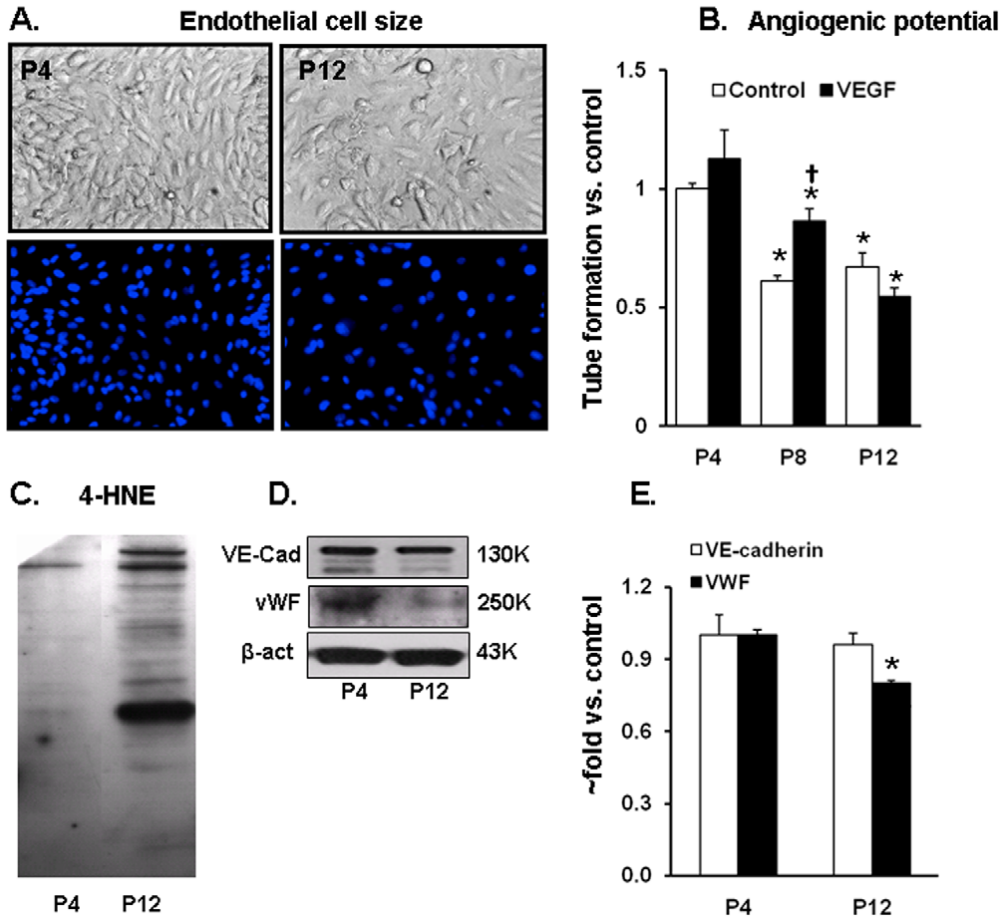
Senescence affects morphology and angiogenic potential of endothelial cells. A. Cell size in late (P12) passage cells is increased by 36% compared to early (P4) passage cells as judged by a number of cells within the field of view in confluent cultures, and the number of nuclei stained with DAPI in 100% confluent cultures is smaller in P12 cells. B. Graph depicts the length of tubes formed by P4, P8 and P12 cells on matrigel in the absence or the presence of 20 ng/ml VEGF. Values are expressed as fold change in relation to unexposed P4 Control. C. Representative Western blots for 4-HNE modified proteins in lysates from P4 and P12 HUVECs. D. Representative Western blots for VE-cadherin and von Willebrand factor, and E. Relative protein content in relation to P4 values; data in mean ± SEM from 3 independent experiments. (*) – p<0.05 compared to P4 cells; (†) – p<0.05 compared to the same passage Control.

The late passage endothelial cells displayed typical morphological features of increased size (+36%, p<0.05) and flattened appearance (Figure 1A).

For evaluation of angiogenic potential, passage 4, 8 and 12 cells (P4, P8 and P12) were plated on matrigel in presence or absence of 20 ng/ml of VEGF (Figure 1B). Older HUVECs (P8 and P12) exhibited significant reduction in basal ability to form tubular networks (−40% and −35% vs. P4 cells respectively, p<0.05). P8 and P12 cells also showed differential reactions to stimulation by VEGF. There was enhancement in tube formation in presence of VEGF (+30% *vs.* unexposed, p<0.05) in P8 cells, whereas P12 cells remained totally unresponsive.

The oxidative stress in late passage cultures was dramatically higher as evidenced by the increase in the content of proteins modified by a product of lipid peroxidation 4-hydroxy- nonenal (4-HNE) (Figure 1 C).

In order to confirm retention of endothelial phenotype, we measured content of endothelial cell-specific molecules VE-cadherin and von Willebrand factor (vWF). VE-cadherin did not change significantly, although P4 cells showed relative excess of VE-cadherin cleavage products. The vWF was also expressed in P12 cells, although compared to younger cultures its content was about 30% (p<0.05) lower (Figure 1 D and E).

**Figure 2:**
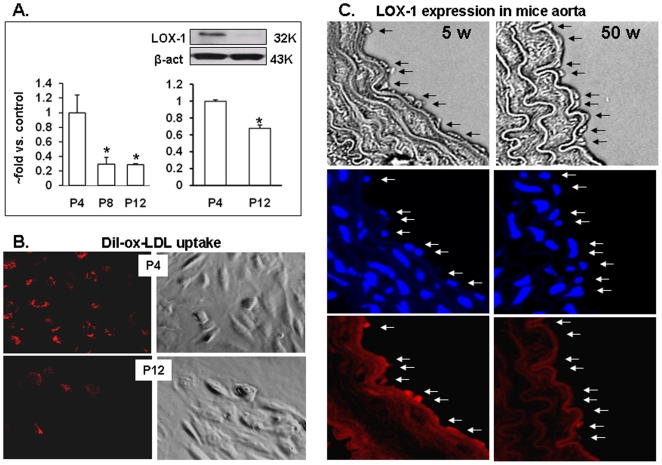
The expression of *LOX-1* in senescent endothelial cells. **A. Changes in** ***LOX-1*** **expression (qPCR, left) and content (right) with senescence.** Data are expressed as fold change in relation to P4 values; data in mean ± SEM from 3 independent experiments. (*) – p<0.05 compared to P4. B. Representative images of Dil-ox-LDL uptake by P4 and P12 endothelial cells. C. LOX-1 immunostaining of aortas from 5- and 50-wk old mice (data representative of 3 separate aortas). Note that the LOX-1 signal in endothelial cells in 50-wk old mice aortas is almost undetectable.

Next, we analyzed the expression of *LOX-1* that has been implicated in virtually all key aspects of atherogenesis, including uptake of ox-LDL, adhesion and transendothelial migration of monocytes, as well as vascular smooth muscle cell proliferation and angiogenesis [10]. There was almost a 4-fold reduction (p<0.01) of mRNA for *LOX-1* accompanied by about 40% decline in protein expression in late passage cells (p = 0.002 vs. P4 cells) (Figure 2A). The observed changes in LOX-1 expression were paralleled by a reduction of Dil-ox-LDL uptake (Figure 2B). Dil-ox-LDL uptake was completely blocked by LOX-1 antibody, in agreement with previous studies [11].

To confirm change in LOX-1 expression with age, we examined LOX-1 expression in aortic sections from young and old mice. As in cultured endothelial cells, age-dependent decline in LOX-1 expression was observed in aortas from 52-week-old (vs. 5 week old) CJ57 mice (P<0.05). As shown in a representative example (Figure 2C), immunostaining for *LOX-1* showed strong signal in endothelial cells from young animals, whereas it was almost indiscernible in the older mice.

**Figure 3:**
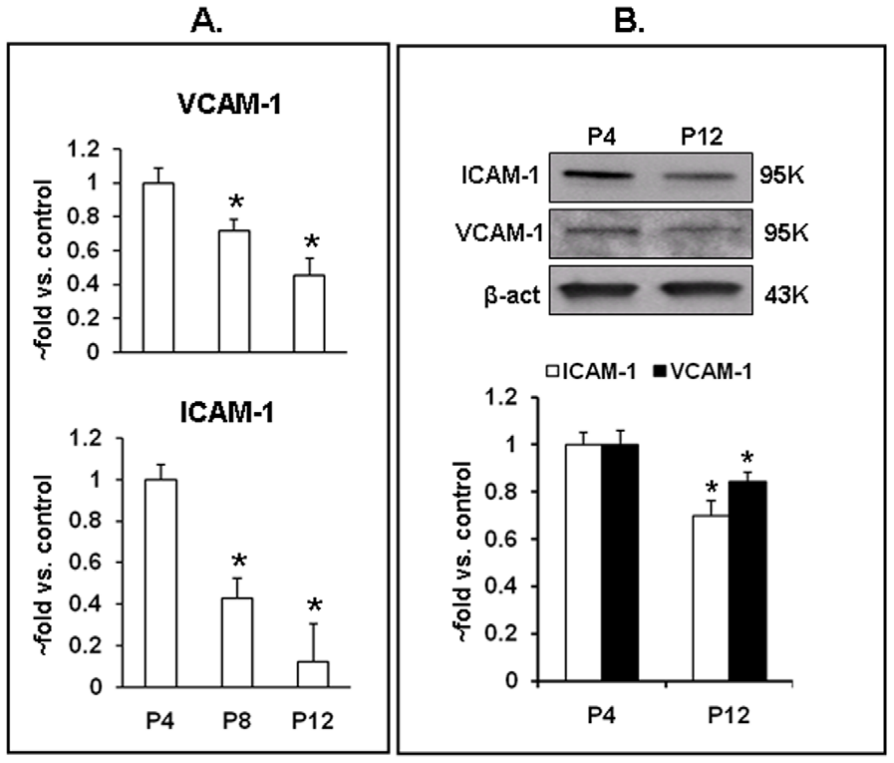
Senescence-dependent decline in *VCAM-1* and *ICAM-1* expression. A. Expression of *VCAM-1* and *ICAM-1* in P4, P8 and P12 cells evaluated by qPCR; B. Protein content for *VCAM-1* and *ICAM-1* evaluated by Western blots. Data from 3 independent experiments are expressed as fold change in relation to P4 values ± SEM. (*) – p<0.05 compared to P4.

*LOX-1* activation has been shown to increase the expression of leukocyte adhesion molecules [12]. In keeping with the *LOX-1* expression data, P8 and P12 cells exhibited a progressive decline of basal transcription for vascular cell adhesion molecule 1 (*VCAM-1*) and intercellular adhesion molecule 1 (*ICAM-1*) (Figure 3A). Changes in expression of these genes were accompanied by a decrease in protein content as well (Figure 3B).

**Figure 4:**
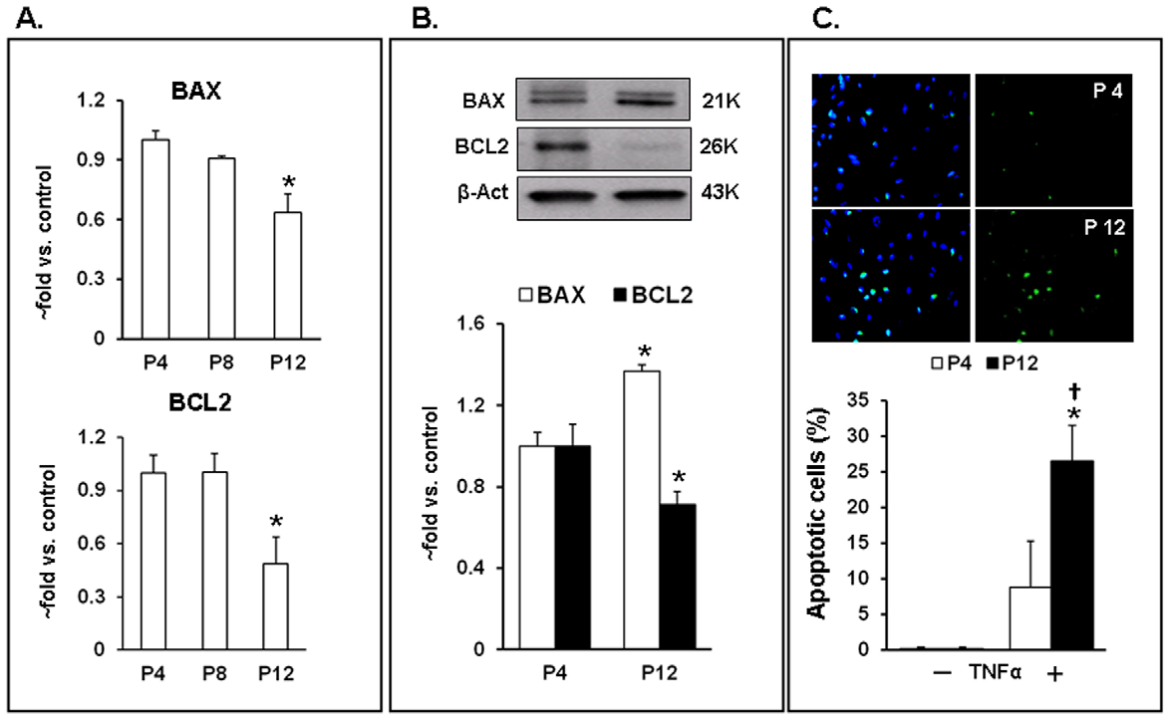
Increased susceptibility to apoptosis in late passage HUVECs. A. Expression of *BAX* and *BCL2* mRNA in P4, P8 and P12 cells evaluated by qPCR; B. *BAX* and *BCL2* protein content evaluated by Western blots. Data from 3–5 independent experiments are expressed as fold change in relation to P4 values ± SEM. C. Apoptosis in HUVECs in response to 24-hr exposure to TNFα (50 µg/ml) measured using polycaspase staining. Upper panel- Representative images of apoptotic cells (green) in P4 and P12 cells treated with TNFα. Lower panel – Quantitation of apoptotic response as a percentage of total cell number. (*) – p<0.05 compared to P4; (†) – p<0.05 compared to the same passage control.

One of the consequences of aging is enhanced susceptibility to apoptosis reported for a variety of cell types [13]–[15]. In our experiments, senescence was associated with a progressive decline in mRNA for both *BAX* and *BCL2*, with a significant change in P12 cells (p<0.05 vs. P4 cells) (Figure 4A). Western blots, however, showed a decline in *BCL2* and an increase in *BAX* in senescent cells (Figure 4B) resulting in almost 3-fold increase in *BAX*/*BCL2* ratio. The discrepancies between changes in *BAX* mRNA and protein content probably reflect enhanced utilization of RNA for translation and/or decrease in protein turnover. We also examined apoptotic response of P4 and P12 HUVECs to 24-hour exposure to TNFα (50 µg/ml). On an average, 9% of P4 cells were positive for polycaspase staining, whereas the number of apoptotic cells in P12 cultures were more than 25% (p<0.01 vs. P4 cells) (Figure 4C).

**Figure 5:**
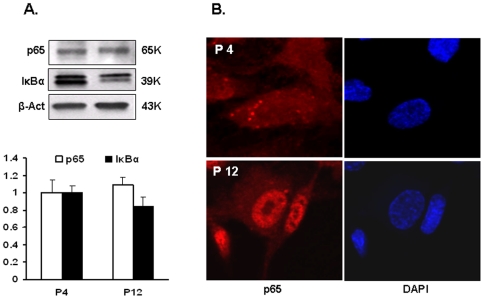
Senescence-dependent changes in sub-cellular localization of NF-kB p65. A. Changes in NF-kB p65 and *IκBα* content in P4 and P12 cells evaluated by Western blots. Data are expressed as fold change in relation to P4 values ± SEM. B. NF-kB p65 immunostaining of P4 and P12 cells. Note that the significant fraction of p65 translocates to the nuclei in P12 cells.

Development of low grade inflammation, characterized by activation of *NF-κB*, is typical for aging. In endothelial cells, exposure to ox-LDL invokes increase in ROS production and *NF-κB* activation; both processes are mediated by *LOX-1* [16]. We did not observe significant changes in the overall content of p65 subunit of *NF-κB* (Figure 5A), but its regulatory counterpart, *IκBα*, tended to decline. However, immunostaining for p65 revealed striking differences in its sub-cellular distribution in older and younger cells. In P12 cells, a considerable fraction of p65 had migrated to the nuclei, whereas in P4 cells the signal was largely limited to the cytoplasm (Figure 5B).

## Discussion

Endothelial senescence has been demonstrated *in vitro* and *in vivo* using several markers such as β-galactosidase staining, telomere shortening, accumulation of oxidative damage and increased expression of p53 and RB [4], [6], [17]–[19]. It is of note that Vasa et al [17] used the same multiple passage of HUVECs s used in the present study. There is, however, a significant disparity in the rate of senescence in the vascular system depending on local parameters of hemodynamic stress and cellular turnover [20], [21].

In this study, we examined endothelial senescence in multiple passages of HUVECs, and show that endothelial senescence is accompanied by a decline in spontaneous and VEGF-stimulated angiogenesis, reduction of *LOX-1* expression, decrease in expression of adhesion molecules and shift to a pro-apoptotic state.

Others have also studied the phenomenon of impairment of angiogenesis with aging. Rivard et al [22] showed that the development of collateral vascularization following femoral artery resection is compromised in old rabbits and mice, and attributed it to reduced NO release and *VEGF* expression. Hoenig et al [23] suggested that the decreased neovascularization potential in the aged is related to depressed *HIF-1* signaling. Our data complement these observations, and suggest that the effects of senescence are somewhat more complex and entail abnormal response to physiological regulators of angiogenesis as well.

Of particular interest is the previously unreported decline in *LOX-1* transcription as endothelial cells age. In the present study, the reduction in *LOX-1* was dramatic in late passage endothelial cells, and this phenomenon was confirmed in the endothelial lining of aortas of old mice. *LOX-1* is a primary scavenger receptor in endothelial cells and plays a significant role in a variety of endothelial functions [24]). Recent studies by our group have implicated *LOX-1* in the stimulation of angiogenesis via activation of NADPH oxidase and consequent increase in ROS production [25], [26]. The role of *LOX-1* in mediation of pro-angiogenic action of small concentrations of ox-LDL and angiotensin II was elucidated utilizing NADPH oxidase inhibitors and *LOX-1* abrogation, and it can be expected, therefore, that depletion of *LOX-1* in the senescent endothelial cells would contribute to depressed angiogenic potential.

We also observed that endothelial senescence was associated with enhanced cell apoptosis which may have implications in reduced angiogenesis. We examined ox-LDL-mediated apoptosis and it was not affected in the P12 cells (data not shown), most likely reflecting a deficit of *LOX-1* which is required for ox-LDL-induced apoptosis [27]. There was, however, a significant increase in TNFα-induced apoptosis in the older endothelial cells. The altered pattern of apoptosis was associated with changes in *BAX* and *BCL2* expression, suggesting that these mediators of apoptosis are also adversely affected by the aging process.

A pro-apoptotic shift appears to be typical of senescence, and increased susceptibility to apoptosis has been reported for variety of cell types including fibroblasts, myocytes, epithelial and endothelial cells [13]–[15], [28]. The reported hypersensitivity of endothelial cells to TNFα by Hoffman et al [28] is similar to our results; however, enhancement of apoptosis in response to ox-LDL observed in the same study is different from our results and counters the expectations based on the decline of *LOX-1* expression in senescent cells. These authors, however, did not provide information on the concentration of ox-LDL used for their experiments which, if beyond the physiological limits, could exert non-specific cytotoxicity.

Activation of *NF-κB* in senescent cells agrees with previous findings [16], [29], and at the same time appears paradoxical in the context of suppression of *LOX-1* and adhesion molecules in the senescent cells. In our studies, expression of *VCAM-1* and *ICAM-1* was significantly suppressed in the P12 cells as compared with P4 cells. Both VCAM and ICAM contain *NF-κB* binding sites in their promoters and are upregulated upon *NF-κB* activation [30]. Previous reports give discordant accounts on adhesion molecules profiles of senescent endothelial cells varying from down-regulation [30] to stimulation [31]. Our results, however, are in line with the microarray data from hearts of *LOX-1* null mice [32] The search of database for *NF-κB* target genes revealed inhibition of a about quarter (79/272) of the gene pool by more than 20% (unpublished data). *VCAM-1* and *ICAM-1* were also found to be downregulated in *LOX-1* null mice transcriptome by 35% (p_two tail_ = 0.0014) and 16% (p_two tail_ = 0.12), respectively. These findings suggest that *LOX-1* may function as an intermediary between *NF-κB* and several of its target genes and, when inhibited as was the case in our senescent cells, partially counteract effects of *NF-κB* activation.

With regard to aging-associated pathologies, some of the observed senescent-driven changes in endothelial cells – for example, compromised angiogenic potential and less permissive environment for cell attachment - could facilitate transition of atherosclerosis to more benign phenotype in elderly. In this context, *LOX-1* emerges as an important senescence factor which may determine the rate of vascular senescence in younger organisms and, at the same time, due to its own aging-associated decline, positively affect the dynamics of disease progression in later stages of life.
